# Methyl glycosides via Fischer glycosylation: translation from batch microwave to continuous flow processing

**DOI:** 10.1007/s00706-018-2306-8

**Published:** 2018-11-17

**Authors:** Jonas Aronow, Christian Stanetty, Ian R. Baxendale, Marko D. Mihovilovic

**Affiliations:** 1 Institute of Applied Synthetic Chemistry, TU Wien, Getreidemarkt 9, 1060 Vienna, Austria; 2 Department of Chemistry, Durham University, South Road, Durham, DH1 3LE UK

**Keywords:** Glycosides, Heterogeneous catalysis, Flow chemistry, Carbohydrates

## Abstract

**Abstract:**

A continuous flow procedure for the synthesis of methyl glycosides (Fischer glycosylation) of various monosaccharides using a heterogenous catalyst has been developed. In-depth analysis of the isomeric composition was undertaken and high consistency with corresponding results observed under microwave heating was obtained. Even in cases where addition of water was needed to achieve homogeneity—a prerequisite for the flow experiments—no detrimental effect on the conversion was found. The scalability was demonstrated on a model case (mannose) and as part of the target-oriented synthesis of d-*glycero*-d-*manno* heptose, both performed on multigram scale.

**Graphical abstract:**

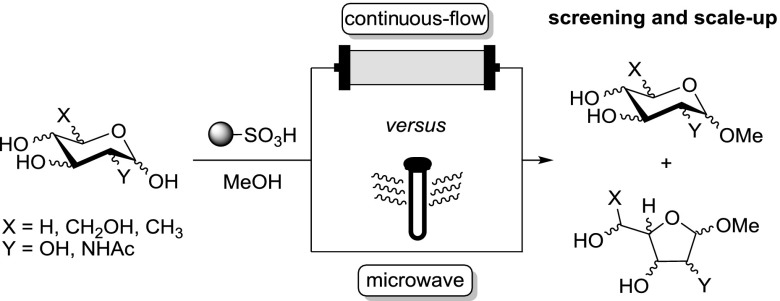

**Electronic supplementary material:**

The online version of this article (10.1007/s00706-018-2306-8) contains supplementary material, which is available to authorized users.

## Introduction

Fueled by the widespread biological importance of glycosides [1, 2], significant research efforts have been invested into their efficient synthesis [3, 4]. Apart from the myriad of sophisticated alternatives [5–7], the Fischer glycosylation, developed in the early 1890s as the earliest glycosylation protocol [8–10], still remains one of the most valuable preparative methods for simple glycosides [4, 11]. Particularly, methyl glycosides serve as popular anomerically protected starting materials for the synthesis of more complex monosaccharide derivatives. In the classical Fischer glycosylation, an alcoholic solution of an unprotected sugar in the presence of a strong acid is heated at reflux to yield the corresponding glycosides [8–10, 12]. Mechanistically, this process initially produces predominantly the furanosides as the kinetic products and only after prolonged reaction time does the equilibrium shift towards the thermodynamically more stable pyranosides (Scheme 1) [13]. To avoid cumbersome post-reaction acid neutralization and the separation of the resultant salt during workup, immobilized acids or acidic ion-exchange resins have been introduced [14–18].

**Figure:**
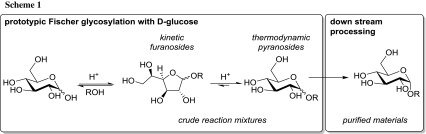

**Fig. 1:**
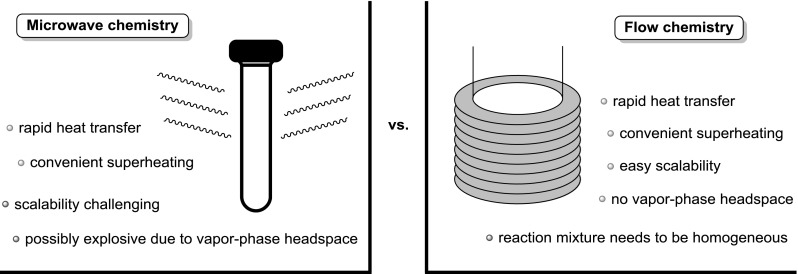
Comparison of microwave and continuous flow chemistry [21]

In 2005, Bornaghi et al. reported on the microwave-acceleration of Fischer glycosylation as a promising approach to overcome the long reaction times required under the classical conventionally heated processes [19]. Following up on this work, Roy et al. successfully demonstrated that montmorillonite K-10 catalyzed Fischer glycosylation under microwave irradiation [12]. However, microwave-mediated procedures suffer from scalability [20, 21], an issue that is often addressed by translating energy intensive chemistry into the flow regime, which generally allows for similar acceleration of reaction rates (Fig. 1) [21–25]. The rate acceleration and concomitant shortening of reaction times in flow reactors are mainly attributed to the fast heat transfer enabled by the high surface area-to-volume ratio as well as higher spatial excess of catalyst in the case of heterogenous catalysis [26]. Additionally, the ease of promoting superheating of reaction mixtures [27] and the absence of a vapor-phase headspace renders flow chemistry a safe and timesaving methodology [21–25]. The downside of this methodology is the general requirement for the avoidance of solids in the flow streams in order to avoid clogging of reactor channels [26].

Although it had been mentioned as a promising scale-up option for corresponding microwave protocols [19], to the best of our knowledge, there has never been a study to test and demonstrate its feasibility. In contrast, more complex glycosylations with glycosyl donors and promotors have already been studied under conditions allowing rapid mixing and efficient removal of heat [28–32].

In the recently reported short synthesis of the l-*glycero*-d-*manno* heptopyranose peracetate **3**, we have utilized such a continuous flow Fischer glycosylation at 100 mmol scale [33]. The Fischer glycosylation step allowed us to circumvent severe reproducibility issues faced in the direct acidic acetylation of the parent bacterial heptose **1** (attributed to the insolubility of **1** in Ac_2_O/AcOH), hampering the reliable production of **3**, in particular at the desired large scale (Scheme 2) [33].

**Figure:**
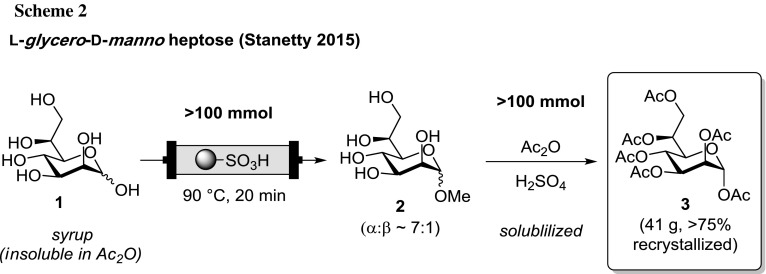

Expanding on our results in this successful case study, we set out to thoroughly and systematically investigate the methyl glycoside formation under Fischer glycosylation conditions in a flow regime.

## Results and discussion

Our aim was a comprehensive comparison of microwave and corresponding continuous flow conditions; therefore, we selected a range of different sugars, including hexoses, pentoses, an acetamido sugar, a deoxy sugar and an uronic acid for our survey. The use of methanol as the acceptor also targeted the minimization of problems associated with heterogeneity within this study. Within preliminary experiments, we exposed the monosaccharides to different reaction conditions using a microwave oven, for two reasons. First, we wanted to be able to reliably identify and quantify all species of interest, particularly the kinetic furanosides usually formed in only minor proportions. Secondly, we wished to compare these results obtained in house under microwave conditions with the corresponding flow-based experiments (see Table 1).

**Table 1:**
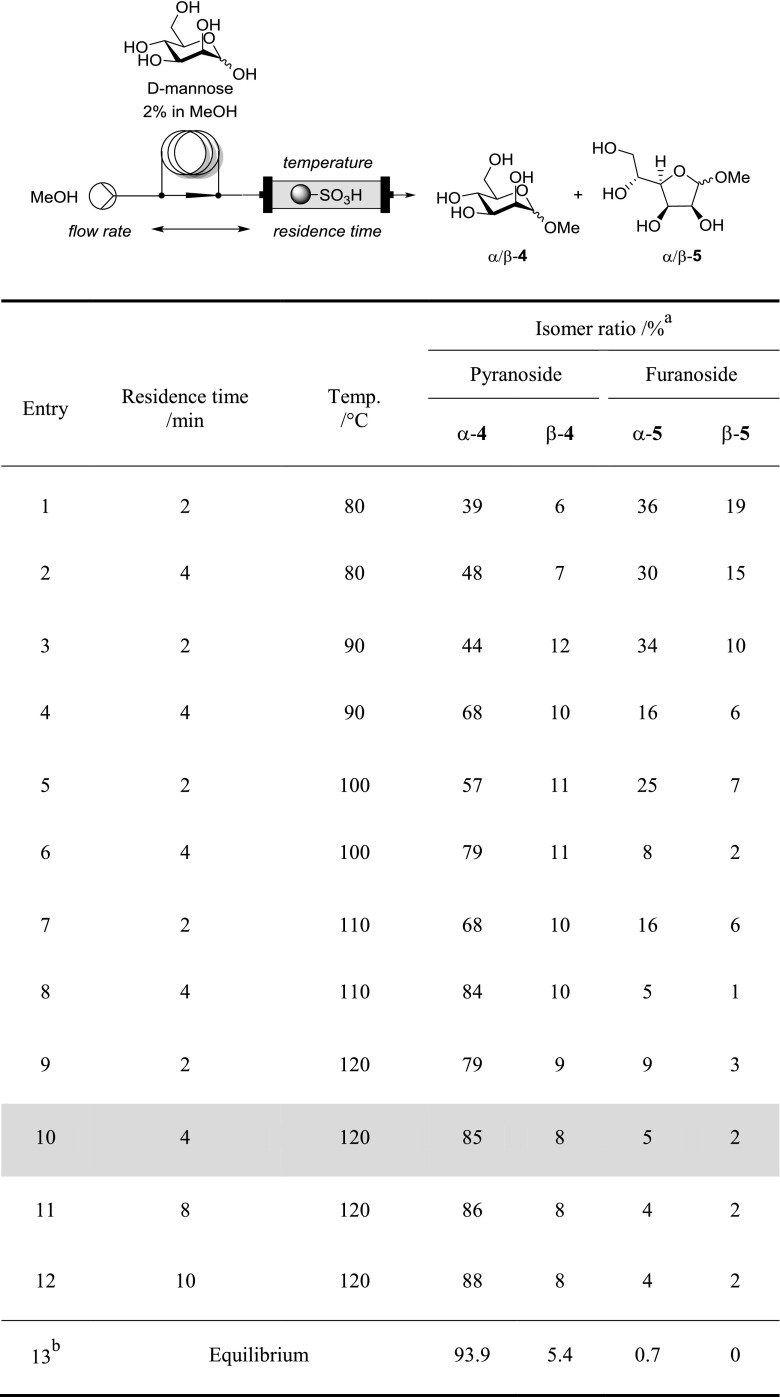
Screening of reaction conditions for the Fischer glycosylation of d-mannose in continuous flow

^a^Determined by ^1^H NMR (for details see Supporting Information)

^b^Mannose (2%) in 1% methanolic hydrogen chloride at 35 °C until composition was constant; no change upon heating to 64 °C (batch process) [11]

For the optimization of the flow process, d-mannose was selected as it features a strong preference for one isomer, the methyl α-pyranoside, under equilibrium conditions [11] which allows for an easier interpretation of how close to the equilibrium conditions a specific data point is. Further, it showed sufficient solubility in pure MeOH. We performed a screen of temperature and residence time by injecting plugs of a mannose stock solution into a bulk MeOH stream passing through a heated column reactor filled with QuadraPure™ sulfonic acid beads (QP-SA). Throughout this study, isomer analysis of the evaporated product streams was performed by ^1^H NMR through integration of diagnostic signals that had prior been assigned via ^1^H, ^13^C, and 2D-NMR experiments and/or comparison to relevant literature data (the diagnostic signals used are compiled in the supporting information).

It is noteworthy that at lower temperatures and/or shorter residence times small amounts of the reducing sugar were still observed (Table 1, entries 1–6). As expected, the composition is shifted towards higher percentage of the pyranosides, particularly the α-pyranoside α-**4**, when employing higher temperature and longer residence times; this correlates with the equilibrium conditions reported in the literature (Table 1, entry 13). However, only a small increase in the α-**4** content was observed at 120 °C with prolonged residence times exceeding 4 min (Table 1, entries 9–12), which were the conditions chosen for the comparative experiments with the other sugars and for comparison with the corresponding microwave (µW) experiments (4 min, 120 °C).

Next, we started the exploration of the monosaccharide scope by passing methanolic solutions of different sugars through the reactor under the optimized conditions, analyzing the product streams analogous to above and comparing the observed data with the microwave experiments (Table 2). For sugars insoluble in MeOH, a minimal amount of water was added until homogeneity at rt was obtained. Pure MeOH remained the bulk solvent which did not lead to any issues with precipitation, likely because dilution in the packed bed reactor was accompanied by heating and the onset of conversion to more soluble glycosides. A high level of consistency was attained between the results in the continuous flow and the microwave-mediated Fischer glycosylation across the series of experiments. Noteworthy, even the addition of water (tentatively shifting the equilibrium towards starting materials) was widely tolerated. Although 7.5 and 1.5% of water had been added to the reaction solutions of d-glucose (Table 2, entry 3) and d-xylose (Table 2, entry 7), the results of these flow experiments were similar and rather closer to the published equilibrium conditions (at reflux conditions) compared to their water-free, microwave-mediated counterparts (Table 2, entries 4 and 8, respectively) [11]. Furthermore, even though d-galactose required the addition of 35% water to create a homogenous phase, the proportions of the resulting glycosides were again closer to those found at equilibrium in pure MeOH (Table 2, entries 5 and 6) [11]. While the applied standard set of conditions were sufficient for most sugars to approach equilibrium within 4 min, the conversion of d-ribose required a longer residence time of 10 min (Table 2, entries 13 and 14) to allow for reasonable comparison of the two regimes at all. Shorter reaction times led to a product mixture containing a large amount of minor, unidentified glycoside species, both in the microwave synthesis and the flow synthesis. Noteworthy, all other reaction mixtures approached the published ultimate equilibrium conditions [11], never exceeded them though, which is in contrast to several reports [12, 19]. The reported ratios of, for example methyl glucopyranosides (up to 15:1 α:β) in the microwave accelerated Fischer glycosylation (compared to 67:33 at equilibrium under the conventional regime) [11] could neither be reproduced in our hands nor are they comprehended by us.

**Table 2:**
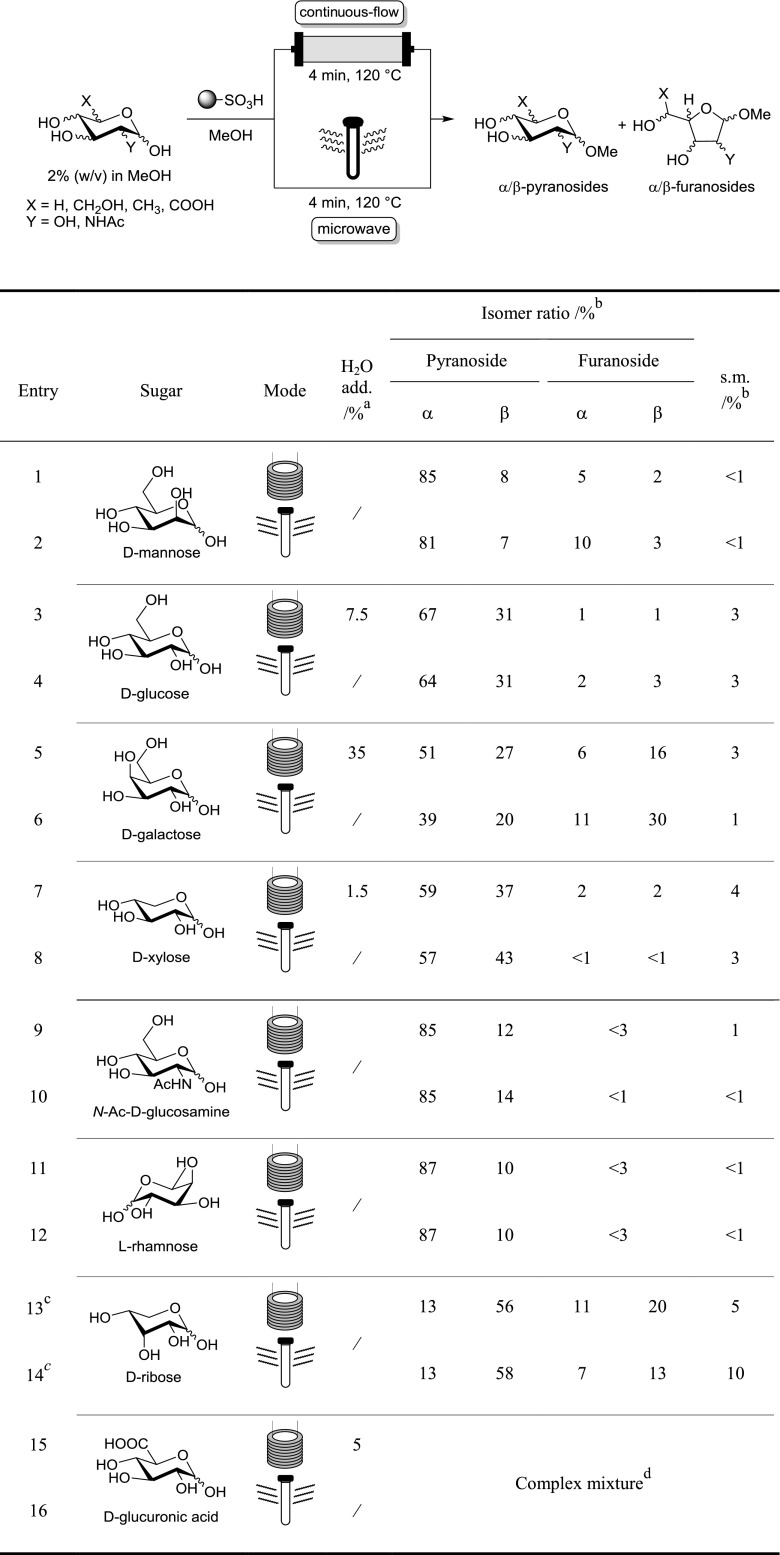
Comparison of the Fischer glycosylation of various monosaccharides under continuous flow and microwave conditions

^a^Refers to %(v/v) water per solution

^b^Determined by ^1^H NMR spectroscopy (for further details see the Supporting Information)

^c^10 min reaction time each

^d^Unidentified product mixture which was not further investigated

## Demonstration of successful upscaling to multigram quantities

Next, we demonstrated the ease of scalability in the formation of methyl mannosides under the optimized conditions (120 °C, 4 min), generating a throughput of 1.2 g/h of crude product for a continuous run of 10 h (Scheme 3). During the processing, an aliquot of the product stream was sampled and analyzed every hour via ^1^H NMR to confirm the steady-state operation and α- and β-pyranoside ratio (α-**4**, β-**4**) which confirmed no detectable decrease in catalyst activity over the entire course of the experiment. Pure methyl α-d-mannopyranoside was obtained from the crude material (12.4 g) by recrystallization from methanol, yielding 9.4 g of the desired product, representing almost four times the mass of catalyst used. This beneficial catalyst to product ratio is one of the major advantages of the flow regime over microwave chemistry, which required at least 300 wt% QP-SA for comparable conversion in a single experiment under equivalent conditions (see the Supporting Information).

**Figure:**
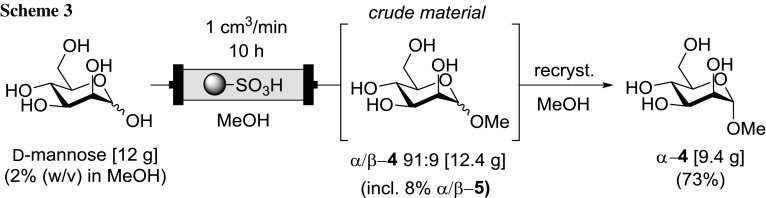

As an additional example of utilization in a synthetic route, we applied our setup and methodology within the established synthetic approach towards d-*glycero*-d-*manno* heptose **9** (Scheme 4) [34, 35], which is the biological precursor of **3**. The key intermediate **5** (derived from d-mannose in multiple steps) underwent OsO_4_ mediated dihydroxylation to deliver dd-*manno*-isomer **6** as a mixture with the minor ll-*gulo* isomer **7** [34, 35]. This crude mixture of **6** and **7** was subjected to conditions analogous to above to achieve simultaneous acetonide cleavage (trans-acetalization) and concomitant trans-glycosylation under continuous flow conditions, thereby locking the targeted pyranose form. The high polarity of **8** allowed for a straightforward separation of all apolar by-products by their extraction into organic solvents in a scalable fashion. Finally, one-pot acetylation and acetolysis analogous to the conversion of **2**–**3** gave the targeted α-pyranose peracetate **9** in pure form as a highly crystalline material in good recovery over the entire sequence on multigram scale.

**Figure:**
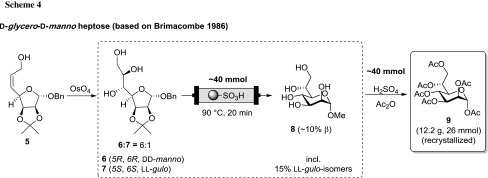

## Conclusion

In the described work, we successfully demonstrated the Fischer glycosylation of various monosaccharides as a continuous flow process with a heterogenous acidic catalyst (QP-SA). High consistency between the ratios of formed products under continuous flow and the related batch-wise microwave conditions was shown. Under the optimized conditions, the addition of water for otherwise insoluble starting materials was tolerated and without detrimental effect on the observed product ratios. The confinement of the catalyst inside the reactor column simplifies downstream processing and allows for increasingly (with scale) better substrate/catalyst ratio in preparative experiments. The developed continuous flow setup offers the possibility of scale-up without any re-optimization which was demonstrated on selected examples.

## Experimental

All starting materials were purchased from commercial sources and used as received. A Biotage Initiator EXP EU Microwave Synthesizer was used for microwave-assisted synthesis. Thin-layer chromatography (TLC) was performed on aluminum sheets precoated with 60 F_254_ silica gel; visualization was accomplished by dipping with anisaldehyde/sulfuric acid and heating. ^1^H and ^13^C NMR spectra were recorded on a Bruker Avance 400 spectrometer or a Varian VNMRS-600. All spectra were recorded at ambient temperature (25 °C). Chemical shifts (*δ*) are quoted in ppm relative to tetramethylsilane and are referenced internally to the residual solvent peaks (CDCl_3_: *δ* = 7.26 ppm; D_2_O: *δ* = 4.79 ppm; MeOH-*d*_4_: *δ* = 3.31 ppm) [36]. Assignments are based on APT, COSY, HSQC, and HMBC spectra. Melting points were measured in open ended glass capillary tubes with a Büchi B-540 melting point apparatus.

### General procedure for the microwave-mediated Fischer glycosylation

A microwave-vial was charged with 30 mg of the sugar, 300 mg QP-SA, and 3 cm^3^ MeOH. The vial was capped and the sample was subjected to microwave irradiation (pre-stirring: 30 s, absorption level setting high) to 80–120 °C for 1–20 min. The reaction mixture was allowed to cool to ambient temperature, was filtered through a small pad of cotton wool and the solvent evaporated. Analysis was performed by ^1^H NMR spectroscopy.

### Flow reactor setup and assembly

For continuous flow reactions, a Syrris Africa flow chemistry module was used for fluid management, fitted with an Omnifit^®^ glass column reactor (100 mm × 10 mm) filled with acidic ion-exchange resin QuadraPure™ SA 450-800 micron (QP-SA; 2.5 g) which was heated in a dedicated aluminum block heated by a stirrer hot plate with associated thermosensor. The outlet flow from the reactor column was connected to a 5 bar back pressure regulator. The void volume of the reactor was measured via differential weighing of the reactor filled with dry QP-SA beads and the reactor filled with QP-SA beads and flooded with MeOH at 22 °C and determined to be 4.0 cm^3^. Consequently, an exemplary flow rate of 1 cm^3^/min equates to a theoretical residence time of 4 min.

### General procedure for the Fischer glycosylation under continuous flow conditions

A methanolic sugar stock solution (2% w/v) was prepared—in case of residual insoluble material, the minimum amount of H_2_O was added and is indicated as v/v % in Table 2 (e.g., 5% H_2_O addition refers to addition of 200 mm^3^ H_2_O to 4 cm^3^ MeOH solution). For the optimization experiments, aliquots of this solution were filled into loops of 1 cm^3^ and were injected at flow rates corresponding to residence times of 2–10 min at 120 °C using methanol as the bulk solvent. The reactor was equilibrated to the conditions by flushing at least three reactor volumes with bulk solvent at the specific conditions, prior to injection. The outlet flow was collected (monitored by TLC) for subsequent NMR analysis.

### Large scale continuous flow preparation of methyl α-d-mannopyranoside (4)

A stock solution of 12 g d-mannose (67 mmol) in MeOH (2% w/v) was pumped through a reactor column at a flow rate of 1 cm^3^/min at 120 °C for 10 h and the outlet flow was fed into a collecting vessel. Every hour of operation a sample of the flow output was evaluated by TLC (CHCl_3_/MeOH/H_2_O 7:3:0.5) and ^1^H NMR analysis. Evaporation of the bulk collected solution yielded 12.4 g of crude solid product (α-**4**/β-**4** = 91:9 + 8% furanosides **2**). Recrystallization from super-heated (100 °C) MeOH in the microwave oven gave colorless needle-shaped crystals upon cooling. After allowing to cool to ambient temperature and storing in the refrigerator (0 °C) overnight the colorless crystalline solid was filtered, washed with a small amount of cold MeOH, and dried in air to give pure methyl α-d-mannopyranoside **4** (9.4 g, 72%). M.p.: 191.1–192.8 °C (MeOH) (lit. 193 °C (EtOH) [37]). Spectral data matched those previously reported [38].

#### d-*Glycero*-α-d-*manno*-heptose hexaacetate (9)

*Fischer trans-glycosylation in flow.* The crude mixture of dd-*manno* and ll-*gulo* triols (maximum total content 40 mmol, **6**:**7** ~ 6:1) [34] was dissolved in 250 cm^3^ MeOH, filtered through a filter paper, and pumped through a packed bed reactor (15 g of QP-SA) at 90 °C with 1 cm^3^/min flow rate. The product solution was evaporated taken up in water and washed with DCM and Et_2_O until all apolar impurities were extracted from the aqueous layer (monitored by TLC). The aqueous layer was evaporated and analyzed by ^1^H NMR indicating a small proportion of remaining acetonide protection. Therefore, the material was taken up in 200 cm^3^ MeOH and passed through the same reactor under identical conditions as before achieving full cleavage of acetonides to methyl heptosides **8**.

*Acetylation and acetolysis.* To the methyl heptoside mixture **8** first 100 cm^3^ Ac_2_O were added and the mixture was stirred for several minutes before 1 g H_2_SO_4_–SiO_2_ [39] was added at rt. The reaction mixture started to warm and within 1 h the reaction mixture turned homogenous. When all material had dissolved, stirring was continued for an additional 30 min to allow the mixture to cool to rt. Then, 3 cm^3^ concentrated H_2_SO_4_ were added dropwise at rt and the reaction mixture was stirred at rt overnight. The reaction mixture was cooled with an ice bath and treated with 32 cm^3^ DIPEA (a change of color from violet to orange, pH ~ 5–7) and was stirred for 10 min before being diluted with EtOAc (200 cm^3^ in total) and washed with water (2 × 200 cm^3^), 1 M HCl (100 cm^3^, pH acidic) and water, NaHCO_3_, and brine, dried over Na_2_SO_4_ and evaporated, co-evaporated from toluene twice and once from EtOH and dried in vacuo to leave a crude material of 19 g of a sticky solid. The material was recrystallized from boiling EtOH (~ 20 cm^3^), crystallization while stirring furnished a colorless solid that was collected by filtration, washed with fresh cold EtOH and hexane to yield, the pure target compound **9** (12.2 g), according to ^1^H NMR with minor amounts of the β-anomer but without any indication for l-*glycero*-l-*gulo* isomers. An analytical sample was prepared by a second recrystallization (500 mg) from boiling EtOH (2 cm^3^, µW, 3 min, 100 °C) to yield large colorless crystals (430 mg) after filtration and washing with fresh cold EtOH. M.p.: 137.1–137.7 °C (EtOH) (lit. 138–139 °C (CHCl_3_) [40]); [*α*]_D_^20^ = + 71 (*c* = 1.0, CHCl_3_) (lit. +66.5 (*c* = 2.5, CHCl_3_) [40]). Spectral data are consistent with those reported [35]. ^1^H NMR (600 MHz, CDCl_3_): *δ* = 6.05 (d, *J* = 2.1 Hz, 1H, H1), 5.36–5.29 (m, 2H, H4, H3), 5.25–5.22 (m, 1H, H2), 5.18 (dt, *J* = 7.0, 3.4 Hz, 1H, H6), 4.41 (dd, *J* = 12.1, 3.6 Hz, 1H, H7a), 4.22 (dd, *J* = 12.1, 7.2 Hz, 1H, H7b), 4.11–4.03 (m, 1H, H5), 2.17, 2.16, 2.10, 2.07, 2.05, 2.01 (6 × s, 6 × 3H, 6 × COCH_3_) ppm; ^13^C NMR (151 MHz, CDCl_3_): *δ* = 170.6, 170.1, 170.0, 169.8 (2 ×), 168.1 (6 × COCH_3_), 90.5 (C1), 72.0 (C5), 70.2 (C6), 68.9 (C3), 68.3 (C2), 66.4 (C4), 61.6 (C7), 21.0, 20.94, 20.88, 20.86 (2 ×), 20.77 (6 × CO**C**H_3_) ppm; HRMS (+ESI–TOF): *m/z* calcd. for C_19_H_26_NaO_13_ ([M +Na]^+^) 485.1271, found 485.1282.

## Electronic supplementary material

Below is the link to the electronic supplementary material.

Supplementary material 1 (PDF 894 kb)
