# Supplementary material for: Methyl glycosides via Fischer glycosylation: translation from batch microwave to continuous flow processing
Source: Monatsh Chem. 2018 Nov 17;150(1):11–9. doi: 10.1007/s00706-018-2306-8 (PMC6320746; doi:10.1007/s00706-018-2306-8)
Supplement: Supplementary file 1 — Supplementary material 1 (PDF 894 kb) [file 706_2018_2306_MOESM1_ESM.pdf]

Supporting Information

Methyl glycosides via Fischer glycosylation – translation from batch microwave to continuous flow processing

Jonas Aronow[1] ● Christian Stanetty[1] ● Ian R. Baxendale[2] ● Marko D. Mihovilovic[1]

[1] TU Wien, Institute of Applied Synthetic Chemistry, Getreidemarkt 9, 1060 Vienna, Austria

[2] Department of Chemistry, Durham University, South Road, Durham, DH1 3LE, United Kingdom

✉ Christian Stanetty

christian.stanetty@tuwien.ac.at

Table S1. Influence of catalyst loading in the microwave-mediated Fischer glycosylation of D-mannose in comparison to the flow conditions (4 min/120 °C).

entry	cat. loading (QP-SA) [wt%]	temp. [°C]	time [min]	isomer ratio [%] (^{1}H-NMR)			
				pyranoside		furanoside	
				α	β	α	β
1	333			65	11	20	4
2	666	120	4	76	11	11	2
3	1000			81	7	10	3
4[a]	<21	120	4	85	8	5	2

[a]Preparative flow experiment with 12 g mannose and 2.5 g QP-SA

Table S2. Overview of the used ^{1}H-NMR (400 MHz) signals of the anomeric protons of methyl glycosides in D_2O (and CD_3OD) and consistent literature references.

glycoside	^{1}H-NMR signal of H-1 of isomeric methyl glycosides [ppm]			
	pyranoside		furanoside	
	α	β	α	β
D-*manno*	4.58 [1]	4.77 [1]	4.95 [2]	4.89 [2]
D-*gluco*	4.81 [1]	4.38 [1]	5.03 [2]	4.90 [2]
D-*gluco (in CD_3OD)*	4.68	4.18	4.93	4.76
D-*galacto*	4.84 [1]	4.32 [1]	4.89 [2]	4.91 [2]
D-*ribo*	4.63	4.68	5.01	4.91
D-*ribo (in CD_3OD)*	4.49	4.61	4.85 [3]	4.75 [3]
D-*xylo*	4.78 [1]	4.32 [1]	5.01 [3]	4.91 [3]
L-*rhamno*	4.70 [4]	4.55 [5]	4.95 [6]	4.88 [6]
N-Ac-D-*glucosamino*	4.77 [7]	4.45 [7]	n.d.	5.13 [8]

Exemplary visual illustrations of the determination of the composition of isomeric methyl glycoside mixtures and residual starting material by NMR analysis

In the following section, three illustrative ^{1}H-NMRs are presented;

a) one representing the simple cases with direct integration of the relevant the H1-signals (D-ribose).
b) one case (D-glucose) where a H1-signal was overlapping with the HDO signal and another signal had to be used as representative diagnostic signals (upon assignment) backed up by measurements in CD_3OD without overlapping of relevant H1 signals.
c) one example (D-xylose) where one species had to be quantified via the signal of the corresponding methoxy-group.

The abbreviations a-f, b-f, b-p, a-p refer to the H1-signals of the corresponding α/β furanosides and pyranosides and R, G, X refers to the H1 signals of the residual reducing sugars (ribose, glucose and xylose) and were based on comparative measurements of authentic samples.

Figure S1. ^{1}H-NMR (400 MHz, D_2O) of the crude mixture of methyl D-ribosides prepared in flow (120 °C, 10 min).

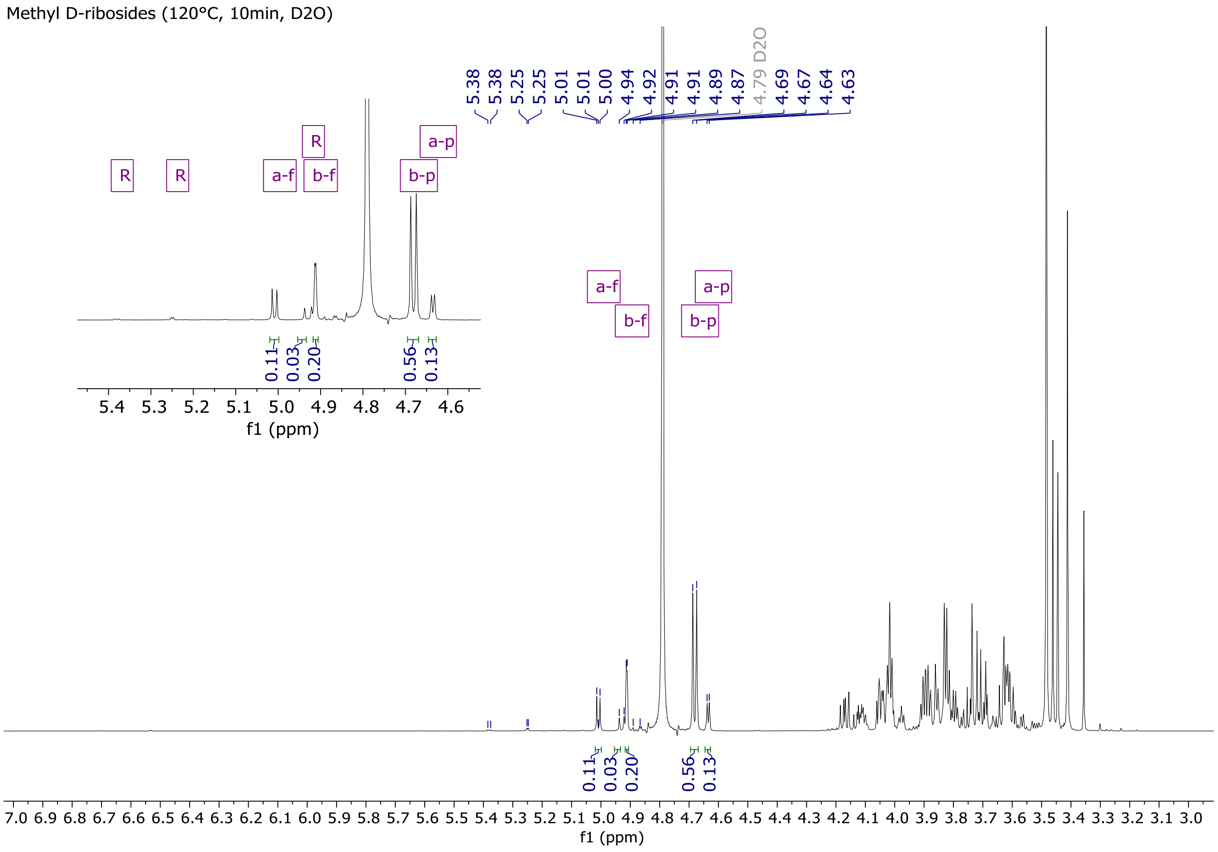

Figure S2. ¹H-NMR of the crude mixture of methyl D-glucosides prepared in flow (120 °C, 4 min) (400 MHz, D_2O, top) and from a corresponding experiment (100 °C, 4 min) furnishing the diagnostic H1 of α-pyranoside and residual glucose (400 MHz, CD_3OD, bottom). Due to the partial overlap of the of the H-1 signal of methyl α-D-glucopyranoside (4.81 ppm) with the D_2O signal (4.79 ppm), this isomer was quantified using the H6a signal at 3.87 ppm, assigned and determined to be free of underlying signals by COSY shown by HSQC. The measurements in CD_3OD furnishing a diagnostic H-1 for the α-pyranoside for direct integration confirmed the validity of the above procedure.

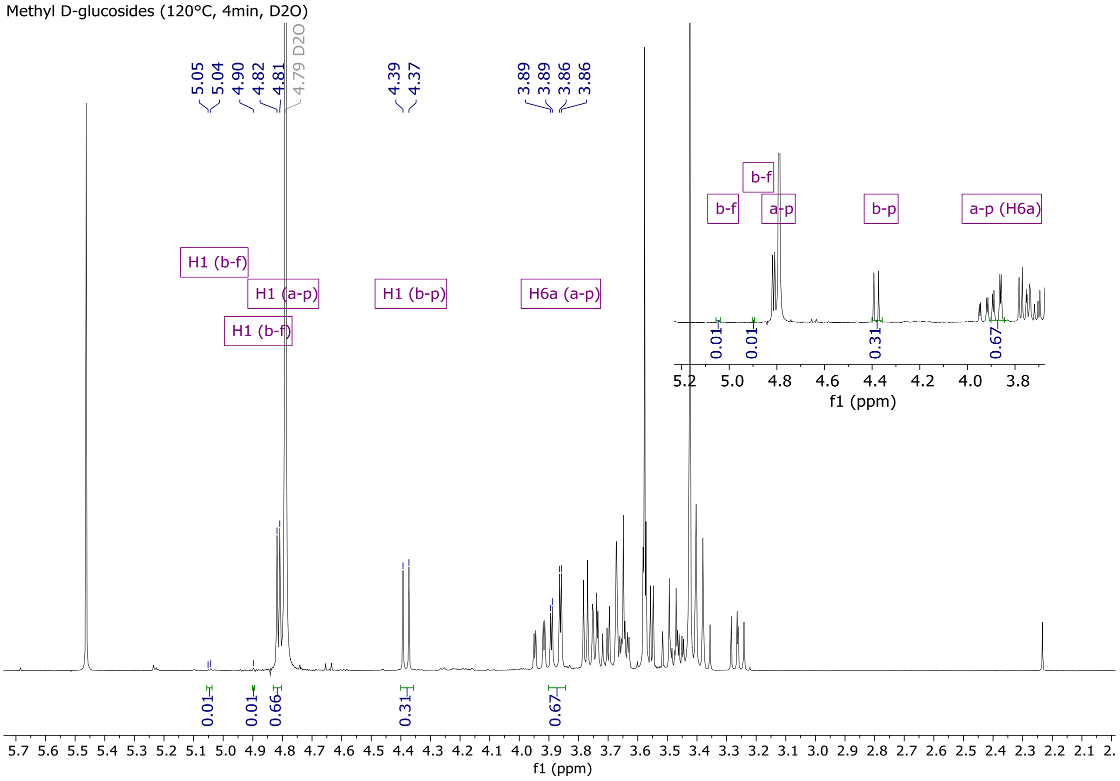

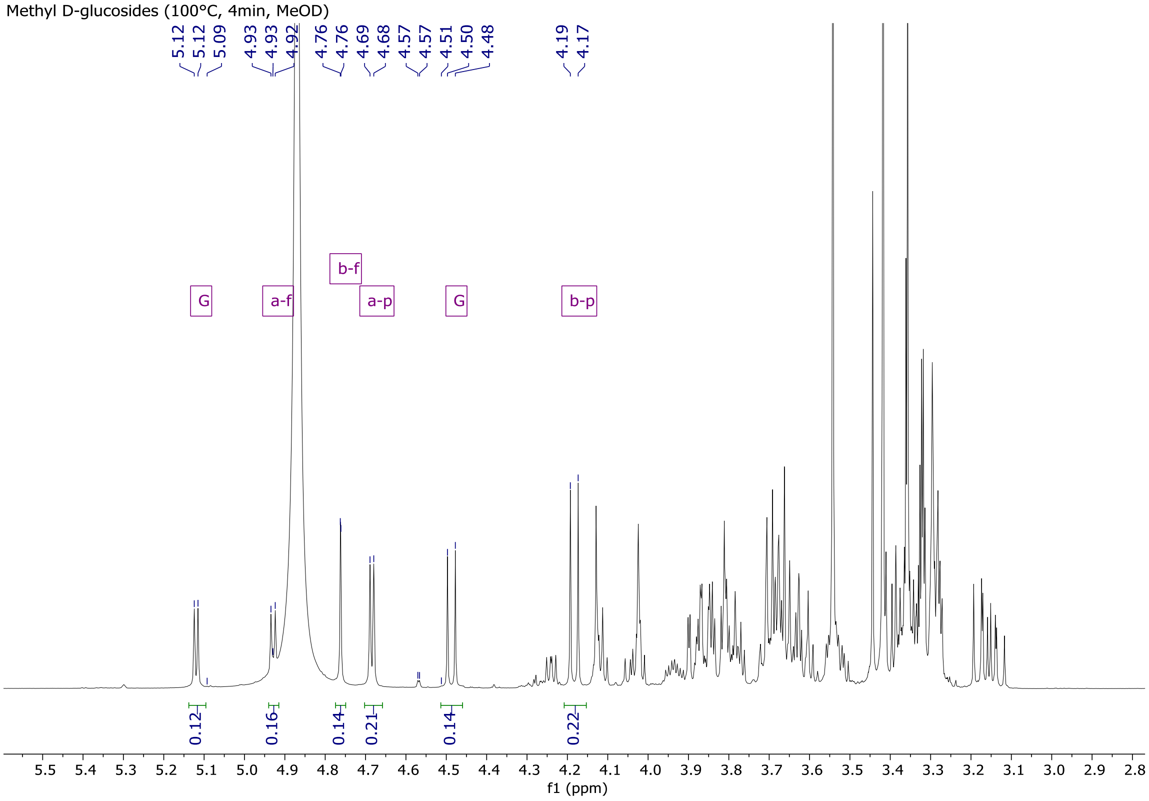

Figure S3. ^{1}H-NMR (400 MHz, D_2O) of the crude mixture of methyl D-xylosides prepared in flow (120 °C, 4 min)

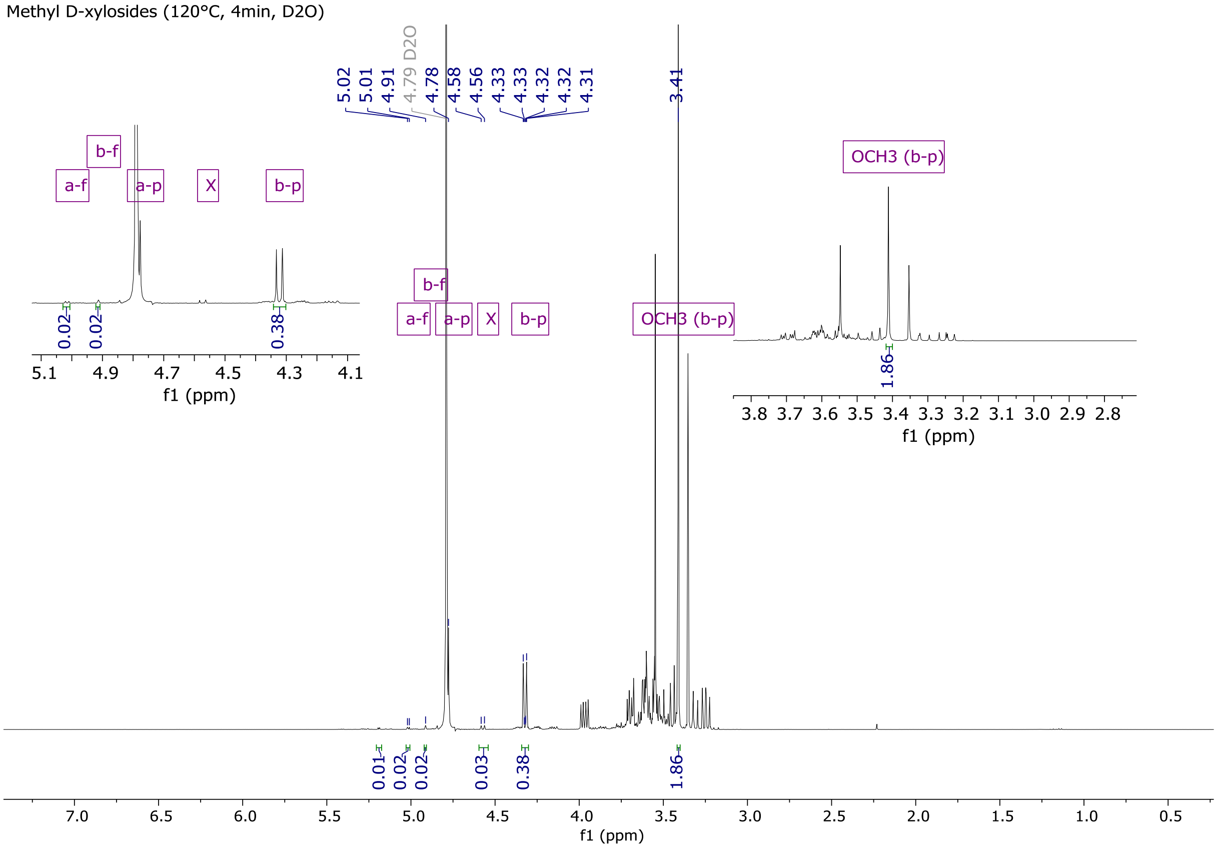

In the case of overlapping of the signal of H-1 of methyl α-D-xylopyranoside (4.78 ppm) with the D_2O signal (4.79 ppm), the isomer was quantified using the alternative signal at 3.41 ppm (representing an 3H-integral). This signal was assigned to the methoxy group of methyl α-D-xylopyranoside *via* 2D-NMR and the absence of any underlying signals was confirmed by HSQC analysis. Noteworthy, the ratios of the main isomers represented in a standard ^{13}C-NMR (Integrals of C1-signals) matched the ones determined via the 1H-NMR (see below) very well.

The percentage of methyl α-D-xylopyranoside was calculated using the following formula:

$$\% = \frac{\left(\text{Integral of signal at 3.41 ppm}/3\right)}{\left(\text{Integral of signal at 3.41 ppm}/3\right) + \text{H1 of }\beta-\text{pyranoside} + \text{H1 of }\alpha-\text{furanoside} + \text{H1 of }\beta-\text{furanoside}} \times 100\%$$

The percentage of the residual isomers was calculated using the following formula:

$$\% = \frac{\text{Corresponding Integral taken from the preceding spectrum}}{\left(\text{Integral of Signal at 3.41 ppm}/3\right) + \text{H1 of }\beta-\text{pyranoside} + \text{H1 of }\alpha-\text{furanoside} + \text{H1 of }\beta-\text{furanoside}} \times 100\%$$

Figure S4 ^{1}H-NMR (600 MHz, $CHCl_3$, top) and ^{13}C-NMR (151 MHz, $CHCl_3$, bottom) of D-glycero-D-*manno*-heptopyranose peracetate (**9**)

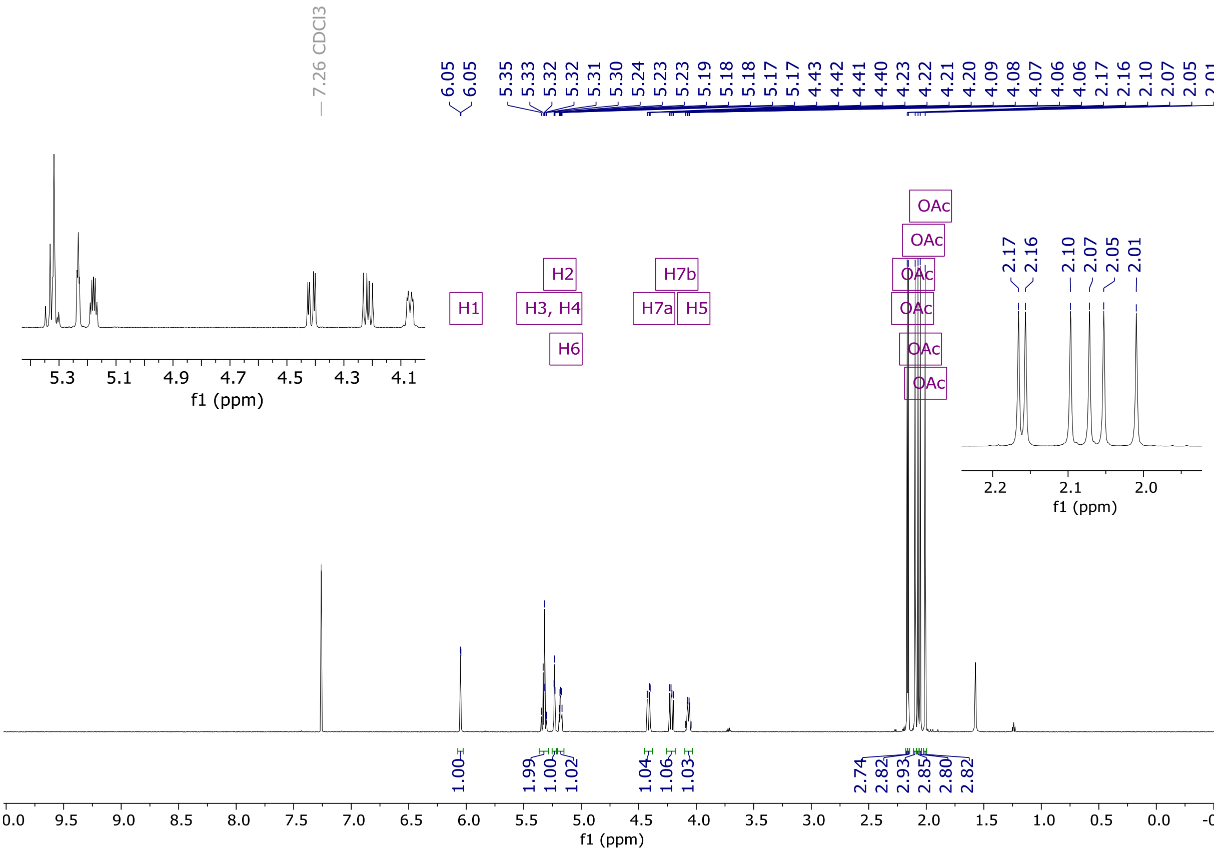

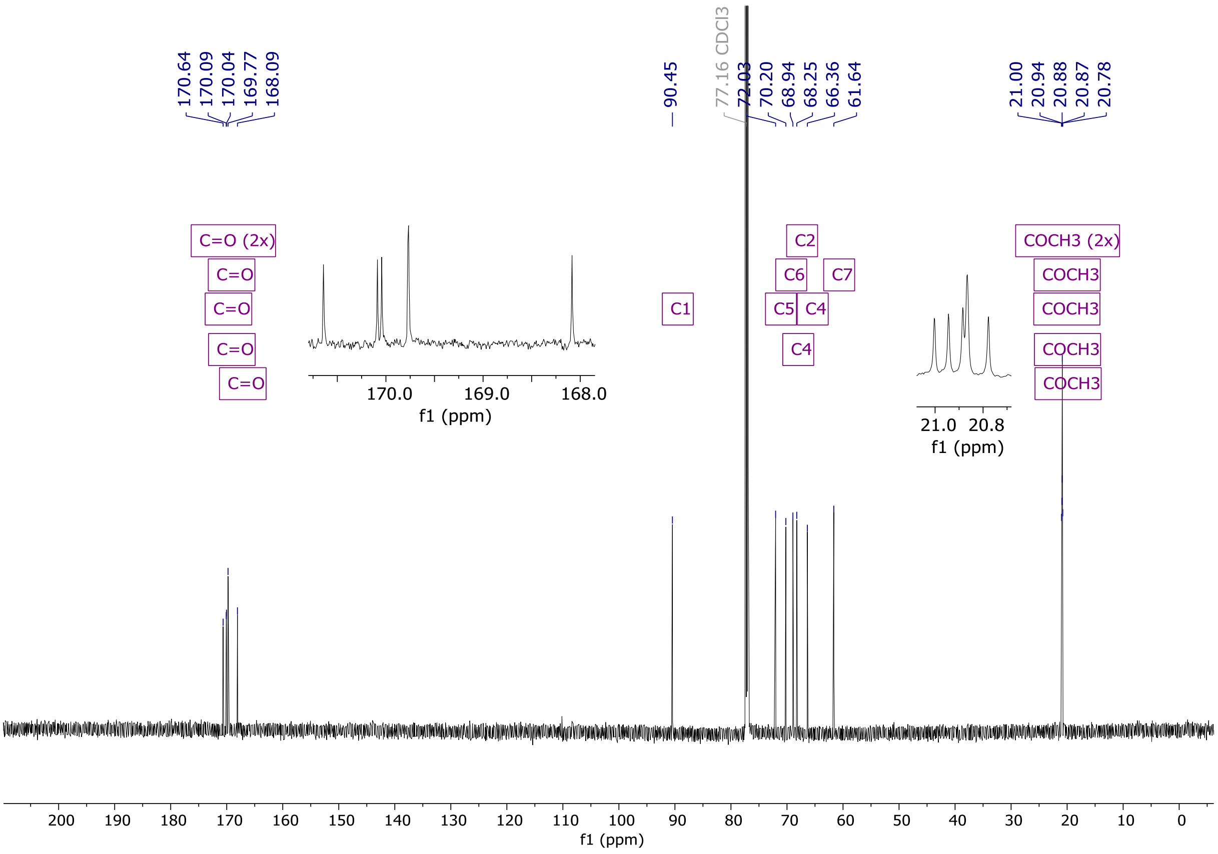

References

1. Podlasek CA, Wu J, Stripe WA, Bondo PB, Serianni AS (1995) J Am Chem Soc 117:8635-8644
2. Angyal SJ (1979) Carbohydr Res 77:37-50
3. Serianni AS, Barker R (1984) J Org Chem 49:3292-3300
4. de Bruyn A, Anteunis M, de Gussem R, Dutton GGS (1976) Carbohydr Res 47:158-163
5. Jansson P-E, Kenne L, Ottosson H (1990) J Chem Soc, Perkin Trans 1:2011-2018
6. Staněk J, Moravcová J, Jarý J (1985) J Carbohyd Chem 4:79-90
7. Zhu Y, Pan Q, Thibaudeau C, Zhao S, Carmichael I, Serianni AS (2006) J Org Chem 71:466-479
8. Furneaux RH, Gainsford GJ, Lynch GP, Yorke SC (1993) Tetrahedron 49:9605-961
